# Improvement of phenolic profile and biological activities of wild mustard sprouts

**DOI:** 10.1038/s41598-024-60452-5

**Published:** 2024-05-08

**Authors:** Hala A. Salah, Alshaimaa M. Elsayed, Roqaya I. Bassuiny, Azza M. Abdel-Aty, Saleh A. Mohamed

**Affiliations:** Molecular Biology Department, National Research Centre, Dokki, Cairo, Egypt

**Keywords:** Mustard seeds, Sprouts, Phenolics, Antioxidant, Antibacterial, Antidiabetic, Biochemistry, Plant sciences

## Abstract

The current study aimed to assess the effect of the germination process of wild mustard seeds on the phenolic profile, antioxidant, antibacterial, and antidiabetic properties, and some relevant enzyme activities. The total phenolic and flavonoid contents increased 5- and 10-fold, respectively, and were maximized on 5-days sprouts. One new phenolic compound was identified on 5-days sprout extract using HPLC. The concentrations of the identified phenolic compounds increased 1.5–4.3 folds on 5-days sprouts compared with dry seeds. The total antioxidant activity multiplied 17- and 21-fold on 5-days sprouts using 2,2-diphenyl-1-picrylhydrazyl** (**DPPH) and 2,2′-azino-bis(3-ethylbenzothiazoline-6-sulfonic acid) assays, respectively. The activity of carbohydrate-cleaving, phenolic-synthesizing and antioxidant enzymes also increased during germination. On 5-days sprouts, there was a substantial correlation between the highest β-glucosidase and peroxidase activities with highest phenolic and flavonoid levels and maximum antioxidant activity. The phenolic extract of 5-days sprouts exhibited antimicrobial activities against *Escherichia coli* and *Staphylococcus aureus* and showed potent antidiabetic activity established by its inhibitory effect against α-amylase and α-glucosidase compared to dry seeds.

## Introduction

The extraction of phenolic compounds from various plant sources has lately emerged as a significant research area because of the health benefits of these compounds^1,2^. Plant phenolic compounds are well known for their range of therapeutic advantages such as anti-inflammatory, antibacterial, antioxidative, anticancer, anti-diabetic, and antivenom^3–9^. Mustard is an annual plant grown in many countries and is a member of the Brassicaceae family. It has several species/varieties such as black, white, brown, wild, rocket, and Ethiopian mustards.The wild mustard (*Sinapis arvensis* L.) is the widest spread species worldwide due to its high fecundity, prolonged seed germination, and natural resistance to several herbicides^10^. Generally, the mustard plant is well-known as an economically important spice that possesses various bioactive compounds^11,12^. Therefore, their seeds also became a valuable source of various bioactive substances with many functional properties. Mustard seeds are high in fat content ranging from 23 to 47%, and glucosinolates with antioxidant, antimicrobial, anticancer and antiherbicides properties^10,13^. Mustard seeds also contained high amounts of valuable antioxidant phenolic compounds such as hydroxybenzoic acid, ferulic acid, and sinapic acid. These valuable antioxidants made mustard seeds could be important to add as food ingredients to protect food against spoilage^10^. Mustard phenolic compounds were previously extracted from some mustard species using different solvents under certain conditions, and the highest antioxidant activity was obtained when using the methanol solution^12,14^. Due to their phenolic content, the addition of ground mustard seeds improved meat products’ color, chemical, microbial, and sensory properties and also prolonged the product’s shelf life^15^. Interestingly, a diet rich in mustard seeds has protective effects against colorectal cancer; besides, some antioxidant enzymes were significantly increased ^16^.

To enhance the biological and nutritive content of plants for human consumption, several processes had been studied. Germination is an effective and low-cost process/method that promotes dynamic and complex changes in the bioactive components and nutritional content of plants^17^. During germination, the quantity and variety of phenolic compounds with potent antioxidant effects increased in a number of medicinal plants, including garden cress seeds and chia seeds. This increase attributed to the rise in some enzyme levels that liberate the conjugated phenolic compounds with complex carbohydrates such as cellulose, in addition to synthesizing new phenolic compounds^18,19^.

Some studies investigated that during germination of white, brown, and black mustard seeds the total phenolic contents and antioxidant activity increased^20,21^. These studies were not deeply address the quantities/varieties of the bioactive phenolic compounds, their various biological activities, and associated enzymes. Therefore, this study aimed to evaluate the germination strategy of wild mustard seeds on the levels of the bioactive phenolic compounds and their antioxidant, antibacterial and antidiabetic properties in comparison to dry mustard seeds. In addition, studying the levels of related enzyme activities (β-glucosidase, peroxidase, catalase, polyphenol oxidase, and phenylalanine ammonialyase), which liberated the conjugated phenolic compounds and synthesizing phenolic compounds.

## Materials and methods

### Chemicals

Guaiacol, catechol, hydrogen peroxide, *p*-nitrophenol-*β*-D-glucopyranoside, L-phenylalanine, trans-cinnamic acid, Folin-Ciocalteu, ABTS (2, 2-azino-bis (3-ethylbenzo-thiazoline-6-sulfonic acid), DPPH (1, 1-Diphenyl-2-picrylhydrazyl), α-amylase and α-glucosidase were purchased from Sigma-Aldrich.

### Seed source

Wild Mustard (*Sinapis arvensis* L.) seeds were purchased from local market, Cairo, Egypt. Experimental research and field studies on the seeds (either cultivated or wild), including the collection of plant material, complied with relevant institutional, national, and international guidelines and legislation.

### Germination process

Mustard dry seeds (10 g) were sterilized in a solution of 0.07% sodium hypochlorite for 5 min at room temperature. They were then rinsed multiple times in distilled water. The seeds were put into a petri dish with wet cotton. The germination of seeds took place in the dark at room temperature (25–30 °C). Every day, distilled water was used to irrigate the seeds. Every day of germination (from 1 to 8 days), the sprouts were collected, dried at 40 °C for 24 h in the oven, and ground.

### Extraction of phenolic compounds

Twenty mL of 80% methanol was added to a flask containing 2 g of powdered mustard seeds or dried sprouts and shaken at 150 rpm for 24 h at room temperature. The extracts were filtered through filter paper Whatman No. 1, and the obtained filtrates were named methanol extracts.

### Determination of phenolics

The phenolics in methanol extract were measured by the method of Velioglu et al.^22^ using the Folin-Ciocalteu reagent. The methanol extract (0.1 mL) was mixed with Folin-Ciocalteu reagent (0.1 mL) and distilled water (0.8 mL) and incubated for 5 min. Then, 20% of sodium carbonate solution (0.5 mL) was added to the mixture and incubated for 20 min. The absorbance was read spectrophotometry at 750 nm. Gallic acid was used as a standard. From the standard curve of gallic acid, the calibration equation is: Y = A + B * X

$${\text{A }} = \, 0.01457;\,{\text{ B }} = \, 0.0619;\,{\text{ R}}^{2} = \, 0.997$$

### Determination of flavonoids

The flavonoids in the methanol extract were measured by the method of Zhishen et al.^23^. The methanol extract (0.25 mL), distilled water (1.25 mL), and 5% NaNO2 (0.075 mL) were mixed and incubated for 5 min. Then add 0.15 mL of 10% AlCl3 and the mixture was also incubated for 5 min. Then 1.0 M NaOH (0.5 mL) and distilled water (0.275 mL) were added. The absorbance was read spectrophotometry at 510 nm. Catechin was used as a standard. From the standard curve of catechin, the calibration equation is: Y = A + B * X

$${\text{A }} = \, 0.00121;{\text{ B }} = \, 0.01232;{\text{ R}}^{2} = \, 0.997$$

### Analysis of phenolic compounds by HPLC

HPLC analysis of phenolic compounds was detected by using Agilent Technologies 1100 series liquid chromatography^24^. The methanol extract was filtered through a 0.45 µm Acrodisc syringe filter before injection. Methanol extract was separated using a 150 × 4.6 µm XDB-C18 column. The column was eluted by acetonitrile (solvent A) and 2% acetic acid (v/v) (solvent B). The total run time was 70 min at flow rate one mL/min with gradient programmed as follows: 100 to 85% (in 30 min), 85 to 50% (in 20 min), 50 to 0% (in 5 min) and 0 to 100% (in 5 min) of solvent B. The obtained peaks were sequentially observed at wave lengths of 280, 320, and 360 nm. The peaks were recognized using identical retention times, UV spectra and compared to commercial phenolic compounds as standards.

### DPPH assay

The DPPH method was used for the determination of antioxidant activity^25^. The methanol extract (0.1 mL) was mixed with 0.9 mM DPPH (0.9 mL) for 30 min incubation in the dark, and spectrophotometry read at 517 nm. The DPPH scavenging % was determined according toequation (1).

1 $${\text{DPPH }}\,{\text{scavenging }}\% \, = \, \left[ {\left( {{\text{O}}{\text{.D}}{. }\,{\text{control}} - {\text{ O}}{\text{.D}}. \, \,{\text{sample}}} \right)/{\text{O}}{\text{.D}}{. }\,{\text{control}}} \right] \, \times \, 100.$$

### ABTS assay

The ABTS method was used for the determination of antioxidant activity^26^. The methanol extract (0.1 mL) was mixed with ABTS reagent (0.9 mL) for 1 min incubation, and spectrophotometry read at 734 nm. The ABTS scavenging % was calculated according to Eq. (2).

2 $${\text{ABTS }}\,{\text{scavenging }}\,\% \, = \, \left[ {\left( {{\text{O}}{\text{.D}}{. }\,{\text{control }} - {\text{ O}}{\text{.D}}{. }\,{\text{sample}}} \right)/{\text{O}}{\text{.D}}{. }\,{\text{control}}} \right] \, \times \, 100.$$

### Total antioxidant activity measurement

According to the equation of Abdel-Aty et al.^18^, the total antioxidant activity was determined according to Eq. (3).

3 $${\text{Total }}\,{\text{antioxidant }}\,{\text{activity}} = {\text{ mg}}\,{\text{ phenolic }}\,{\text{content}}/{\text{mg }}\,{\text{IC}}_{50} .$$

IC_50_ value is the concentration of phenolic compounds required to scavenge 50% of either DPPH or ABTS free radicals.

### Extraction of enzymes

One g of mustard seeds or sprouts was extracted in 50 mM Tris–HCl buffer, pH 7.2. Then, for 10 min, the samples were centrifuged at 10,000 rpm and 4 °C. Each obtained supernatant was named acrude enzyme extract.

### Peroxidase assay

The peroxidase activity (EC 1.11.1.7) was detected according to Miranda et al.^27^ procedure. The reaction mixture containing in one ml: crude enzyme extract (0.1 mL), 40 mM guaiacol, and 8 mM H_2_O_2_ and 20 mM sodium acetate buffer, pH 5.5. The reaction mixture was incubated for one min at room temperature and spectrophotometry read at 470 nm. The increase one O.D. is considered one unit of enzyme activity.

### Catalase assay

The catalase activity (EC 1.11.1.6) was detected according to Bergmeyer procedure^28^. The reaction mixture containing in one ml: crude enzyme extract (0.1 mL), 25 mM H_2_O_2_ and 20 mM sodium phosphate buffer, pH 6.8. The reaction mixture was incubated for one min at room temperature and spectrophotometry read at 240 nm. The decrease 0.1 O.D. is considered one unit of enzyme activity.

### Polyphenol oxidase assay

The polyphenol oxidase activity (EC 1.14.18.1) was detected according to Jiang et al. procedure^29^. Crude enzyme extract (0.1 mL) and 20 mM catechol are all present in 1 mL of the reaction mixture at pH 6.8 using sodium phosphate buffer. The reaction mixture was incubated for 5 min and spectrophotometry read at 400 nm. The increase 0.1 O.D. is considered one unit.

### *β*-Glucosidase activity assay

The *β*-Glucosidase activity (EC 3.2.1.21) was detected according to Gunata et al.^30^ procedure. The reaction mixture containing in one mL: crude enzyme extract (0.1 mL), 0.9 mM *p*-nitrophenyl-*β*-D-glucopyranoside and 20 mM sodium acetate buffer, pH 5.5. The reaction mixture was incubated for 20 min and spectrophotometry read at 405 nm. The release of one µmol *p*-nitrophenol is considered one unit.

### Phenylalanine ammonia lyase activity assay

The phenylalanine ammonia lyase activity (EC 4.3.1.24) was detected according to Goldson et al.^31^ procedure. The reaction mixture containing in one mL: crude enzyme extract (0.1 mL), 40 mM L-phenylalanine and 20 mM Tris–HCl buffer, pH 8.8. The reaction mixture was incubated for 30 min and spectrophotometry read at 290 nm. The release of one µmol trans-cinnamic acid is considered one unit.

### Antimicrobial activity

#### Bacterial strains

Two bacterial strains were used: one Gram-negative *Escherichia coli* (ATCC 51,659) and one Gram-positive *Staphylococcus aureus* (ATCC 13,565).

#### Agar diffusion method

The agar diffusion method was performed according to Bauer et al.^32^ procedure. The antibacterial activity of mustard seeds and 5-days sprout phenolic extracts against pathogenic bacteria was detected on Mueller–Hinton agar. A total 100 µl of each tested bacterium suspension (10^8^ CFU/ml) was spread on the surface of the plates. The tested extracts (50 µg gallic acid equivalent (GAE)) were added to wells which punched by a well borer in the agar medium and incubated for 18 h at 37 ± 1 °C. The antimicrobial activity was evaluated by measuring the clear growth-inhibition zones (mm). Gentamicin is used as a positive control.

#### Determination of minimum inhibition concentration (MIC)

The MIC values for the tested extracts were determined using the agar dilution diffusion technique. 10^8^ CFU/ml of each pathogenic bacterium were put on Muller Hinton agar media and incubated at 37 ± 2 °C for 18 h with various doses of each extract. A MIC value was defined as the lowest extract concentration at which each bacterium’s growth was inhibited.

### In-vitro antidiabetic assays

#### α-Amylase inhibition assay

α-Amylase inhibition activities of mustard seeds and 5-days sprout phenolic extracts were determined as described by Liu et al.^33^. In one mL reaction mixture: 5 units of pancreatic α-amylase were mixed with 20 mM sodium phosphate buffer (pH 7.2) and 0.01 mL phenolic extract or acarbose. After incubation for 5 min at 37 °C, 1% starch (0.1 mL) was added and incubated for 30 min. Then 0.5 mL of dinitrosalicylic reagent was added and boiled for 10 min, and spectrophotometry read at 540 nm. The inhibition was calculated according to Eq. (4).

4 $${\text{Inhibitory }}\,{\text{effect }}\left( \% \right) \, = \, \left( {{\text{O}}{\text{.D}}{. }\,{\text{control }}{-}{\text{ O}}{\text{.D}}{. }\,{\text{sample}}} \right)/{\text{O}}{\text{.D}}{. }\,{\text{control }} \times \, 100$$

IC_50_ value is the phenolic concentration required to inhibit 50% of enzymatic activity.

#### α-Glucosidase inhibition assay

α-Glucosidase inhibition activities of mustard seeds and 5-days sprout phenolic extracts were assayed as reported by Zhang et al.^34^. In one mL reaction mixture: one unit of α-glucosidase was mixed with 0.01 mL of phenolic extract or acarbose at pH 6.8 using sodium phosphate buffer and incubated for 5 min at 37 °C. Then, 2 mM *p*-nitrophenyl-α-glucopyranoside was added, and spectrophotometry read at 405 nm after 15 min of incubation. The inhibition was calculated according to Eq. (5).

5 $${\text{Inhibitory }}\,{\text{effect }}\left( \% \right) \, = \, \left( {{\text{O}}{\text{.D}}{. }\,{\text{control }}{-}{\text{ O}}{\text{.D}}{. }\,{\text{sample}}} \right)/{\text{O}}{\text{.D}}{. }\,{\text{control }} \times \, 100$$

IC_50_ value is the phenolic concentration required to inhibit 50% of enzymatic activity.

All experimental procedures were carried out in compliance with relevant guidelines.

### Statistical analysis

Data were analyzed using one-way ANOVA followed by Tukey’s post hoc test; these tests were conducted in GraphPad Prism version 5. Data are presented as means ± SD (n = 4) and differences were considered significant at *P* < 0.01.

## Results and discussion

Recent research demonstrated that a variety of beneficial phenolic compounds were significantly generated during germination^18,19^. Table 1 showed the phenolic and flavonoid contents of wild mustard seeds during germination. The total phenolic and flavonoid levels of raw mustard seeds (3.62 mg GAE/g and 0.5 mg CE/g, respectively) considerably increased and maximized on 5-days sprouts (18.23 mg GAE/g and 4.9 mg CE/g, respectively) by 5 and 10-fold, respectively, and then gradually decreased until the 8-days of germination (7.2 mg GAE/g and 0.89 mg CE/g, respectively). Bors et al.^20^ found that the total phenolic content of brown and black mustard seeds after 7 days of germination increased from 7.7 to 11.5 mg GAE/g and from 9.3 to 16.8 mg GAE/g, respectively. Also, Rasera et al.^21^ observed that during the germination process of white mustard, there was a positive effect on the total phenolic content.The differences in phenolic contents may be attributed to different mustard cultivars, cultivation regions, and germination conditions. Table 1 shows the ratio of total flavonoid content to total phenolic content (CE/GAE %), which increased during germination and maximized on 5-days sprouts (26.8%).These flavonoids had several bioactive properties such as antioxidant and anticancer effects as reported by Fraga et al.^35^. Liu et al.^36^ reported that during the seed germination the carbohydrates, proteins and cell wall conjugated-phenolic compounds are degraded and lead to increase the simple sugars, free amino acids and free/soluble phenolic compounds. This examination could clarify the increase of total phenolic and flavonoid contents in mustard sprouts.The decline in the total phenolic content of mustard sprouts after 5 days of germination may be attributed to convert of some free phenolic compounds to bound phenolic compounds and/or they consumed in lignin synthesis^37–40^.

**Table 1:** Screening of total phenolic and flavonoid contents of the wild mustard seeds during germination.

Days	Total phenolic mg GAE/g DW	Total flavonoid mg CE/g DW	CE/GAE (%)
0 (seeds)	3.62 ± 0.15^a^	0.50 ± 0.03^a^	13.4
1	5.71 ± 0.35^b^	0.86 ± 0.05^b^	15.0
2	7.46 ± 0.38^c^	1.71 ± 0.10^c^	22.9
3	12.16 ± 0.75^d^	2.85 ± 0.13^d^	23.4
4	16.22 ± 0.92^e^	4.0 ± 0.25^e^	24.6
5	18.23 ± 1.2^f^	4.9 ± 0.27^f^	26.8
6	12.1 ± 0.65^d^	2.3 ± 0.12^g^	19.0
7	9.2 ± 0.15^ g^	1.24 ± 0.08^h^	13.47
8	7.2 ± 0.28^c^	0.89 ± 0.03^b^	12.3

*GAE* gallic acid equivalent, *CE* catchin equivalent. Values are presented as means ± SD (n = 4). Values in the same column with different superscripts (^a,b,c,d,e,f,g,h^) are significantly different at (*P* < 0.01).

Table 2 lists the phenolic compounds of dry mustard seeds and their 5-days sprouts using the HPLC-analysis technique. Thirteen phenolic compounds were identified in the mustard dry seeds, with concentrations ranging from 0.04 to 0.80 mg/g for chrysin and sinapic acid, respectively. Whereas, fourteen phenolic compounds were identified in 5-days mustard sprouts extract, with concentrations ranging from 0.12 to 3.4 mg/g for *p*-coumaric and sinapic acids, respectively. One new phenolic compound (vanillic acid) appeared on 5-days mustard sprouts. During germination, new phenolic compounds are synthesized or transformed^18^. Fifteen phenolic compounds were detected in chia dry seeds extract and seventeen phenolic compounds were detected in their 7-days sprouts^19^. Also, two new phenolic compounds appeared in the garden cress 6-days sprouts^18^.The concentrations of phenolic compounds increased several folds (1.5–4.3) in 5-days mustard sprouts compared to the mustard dry seeds. The *p*-hydroxybenzoic and sinapic acids were the highest phenolic acids in both mustard extracts compared to other detected phenolics. The phenolic profile and antioxidant capacity of white mustard seeds showed high *p*-hydroxybenzoic and sinapic acid concentrations^16^. These phenolic compounds have significant biological properties, including antimicrobial, antibacterial, antidiabetic, anticancer, and antioxidant activity^41,42^. In addition, the concentrations of the identified flavonoids increased in 5-days mustard sprouts compared to mustard dry seeds. This examination may clarify the increase in the percent of flavonoids/phenolics from 13.4% for mustard dry seeds to 26.8% for 5-days mustard sprout as shown in Table 1. Therefore, the phenolic extract of 5-days mustard sprouts could be used as dietary supplements for the prevention of several diseases.

**Table 2:** HPLC analysis of phenolic compounds of wild mustard dry seeds and 5-days sprouts.

Compound	RT	Dry seeds mg/g DW	Day 5 sprouts mg/g DW	Fold increase in sprout
Gallic	5.7	0.52^a^	1.0^b^	1.5
Protocatechuic	9.9	0.09^a^	0.2^b^	2.2
*p*-hydroxybenzoic	15.1	0.70^a^	2.8^b^	4.0
Catechin	18.6	0.42^a^	1.4^b^	3.3
Chlorogenic	20.6	0.31^a^	1.0^b^	3.2
Caffeic	21.4	0.53^a^	1.8^b^	3.4
Syringic	23.0	0.5^a^	1.5^b^	3.0
Vanillic	24.8	ND	0.20	–
Ferulic	32.4	0.28^a^	0.68^b^	2.4
Sinapic acid	33.8	0.80^a^	3.4^b^	4.3
*p*-coumaric	37.2	0.05^a^	0.12^b^	2.4
Rosmarinic	40.0	0.09^a^	0.24^b^	2.6
Cinnamic	42.8	0.07^a^	0.18^b^	2.5
Chrysin	52.0	0.04^a^	0.14^b^	3.2

*RT* retention time, *ND* not detection. Values are presented as means ± SD (n = 4).

Values in the same column with different superscripts (^a,b^) are significantly different at (*P* < 0.01).

Table 3 showed the antioxidant activity during the germination of wild mustard seeds using the DPPH assay. A low IC_50_ (phenolic concentration required to scavenge 50% of DPPH-free radicals) indicates a high antioxidant activity. The recorded IC_50_ extensively decreased from wild mustard seeds (0.056 mg GAE/ml) to their 5-days sprouts (0.016 mg GAE/ ml). In addition, the total antioxidant activity extensively increased (*P* < 0.01) from mustard dry seeds (64) to reach the maximum in 5-days sprouts (1139) by 17-fold. Then, the total antioxidant activity declined until the 8-days after germination. The antioxidant activity of wild mustard seeds during germination using the ABTS assay was also studied and presented in Table 3. The recorded IC_50_values using ABTS considerably decreased from raw mustard seeds (0.015 mg GAE/ml) to the 5-days mustard sprouts (0.0036 mg GAE/ml). And then, the obtained IC_50_ values of ABTS gradually increased up to 8-days sprouts. Also, the total antioxidant activity significantly increased (*P* < 0.01) from seeds (240) to reach the maximum on 5-days sprouts (5063) by 21-fold, followed by a decrease until 8-days of germination. Rasera et al.^21^ observed that extracts obtained from white mustard during germination had a positive influence on the DPPH and ABTS-radical scavenging activity. The edible seed sprouts showed highest antioxidant activity, where the antioxidant activity increased in jack bean 4-days sprouts (~ 1.5-fold)^43^, soybean 7-days sprouts (~ 1.25-fold), and mung bean 7-days sprouts (~ 1.6-fold)^44^. Additionally, Abdel-Aty et al.^19^ showed that the lowest value of IC_50_ and the highest total antioxidant activity using DPPH and ABTS-radicals were detected on 7-days chia sprouts. The antioxidant activity may have increased because mustard sprouts contained higher levels of antioxidant phenolics and flavonoids in addition to the new phenolic acid that was produced.

**Table 3:** The antioxidant activity of the wild mustard seeds during germination using the DPPH and ABTS assays.

Days	DPPH		ABTS	
	IC_50_	Total antioxidant activity	IC_50_	Total antioxidant activity
0 (seeds)	0.056 ± 0.002^a^	64 ± 2.15^a^	0.015 ± 0.001^a^	240 ± 12^a^
1	0.023 ± 0.001^b^	248 ± 8.8^b^	0.0063 ± 0.0003^b^	906 ± 33^b^
2	0.019 ± 0.0012^c^	392 ± 12.7^c^	0.0052 ± 0.0002^c^	1434 ± 66^c^
3	0.018 ± 0.001^c^	675 ± 18.9^d^	0.0045 ± 0.0003^e^	2702 ± 98^e^
4	0.017 ± 0.0012^c^	954 ± 24.8^e^	0.0042 ± 0.0003^e^	3861 ± 122^f^
5	0.016 ± 0.001^d^	1139 ± 35.7^f^	0.0036 ± 0.0002^f^	5063 ± 133^g^
6	0.020 ± 0.0015^c^	605 ± 22.8^d^	0.0046 ± 0.0003^e^	2630 ± 105^e^
7	0.023 ± 0.0015^b^	400 ± 6.7^c^	0.0056 ± 0.0004^c^	1642 ± 88^c^
8	0.025 ± 0.018^b^	288 ± 5.6^b^	0.0060 ± 0.0004^b^	1200 ± 66^c^

IC_50_: mg GAE/ml, Total antioxidant activity: mg phenolic content/IC_50_. Values in the same column with different superscripts (^a,b,c,d,e,f,g^) are significantly different at (*P* < 0.01).

**Figure 1:**
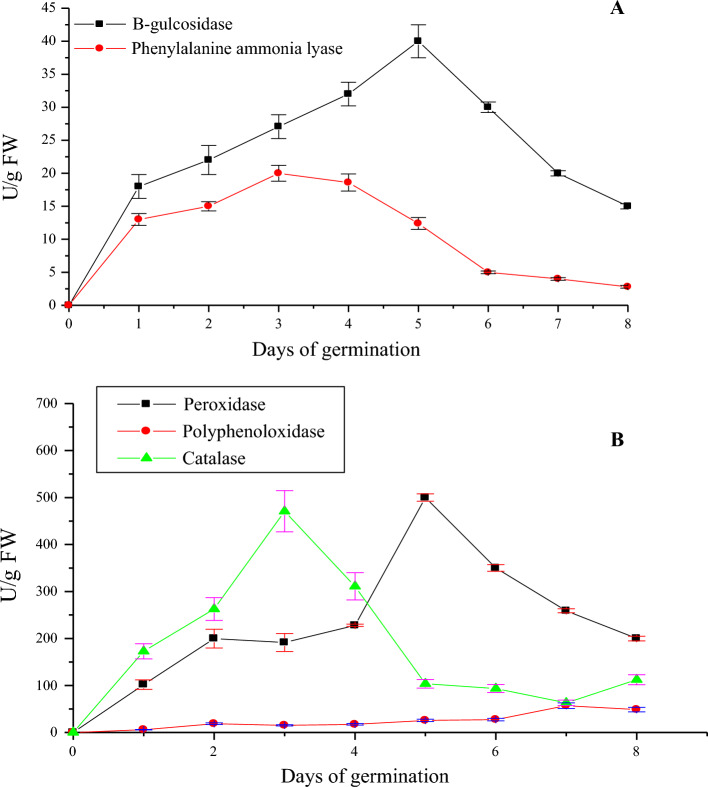
The activity of (**A**) *β*-glucosidase and phenylalanine ammonia lyase and (**B**) polyphenoloxidase, catalase, and peroxidase during the germination of wild mustard seeds. Values were significantly different at (*P* < 0.01).

Some enzymes of plant hydrolyzed macromolecules to produce many bioactive compounds, which improved its functional properties^45,46^. Figure 1A showed a screening of the enzymatic activities of β-glucosidase as carbohydrate-cleaving enzymes and phenylalanine ammonia lyase as phenolic-synthesizing enzymes during the germination of wild mustard seeds. The activity of β-glucosidase increased gradually to reach a maximum on 5-days sprouts (40 U/g) and decreased gradually until 8-days of germination (12.5 U/g). The highest activity of β-glucosidase on 5-days sprouts indicated that there is a high correlation between this enzyme and the highest phenolic and flavonoid contents on the same day. Kranz et al.^47^ observed that the activity of β-glucosidase increased during the germination of barley to reach its maximum activity on 12-days sprouts (102 U/kg) and the activity of β-glucosidase increased during the germination of wheat to reach its maximum activity on 7-days sprouts (400 U/kg). The β-glucosidase efficiently hydrolyzed the phenolic glycosides to release free phenolic compounds^48^. Additionally, the activity of phenylalanine ammonia lyase (PAL) increased gradually to reach the maximum on 3-days sprouts (17.5 U/g) and also decreased gradually until 8-days sprouts (7 U/g). However, the activity of PAL showed a low correlation with the highest phenolic and flavonoid contents and the antioxidant activity on 5-days sprouts. Zhan et al.^49^ investigated the activity of PAL during the germination of garden cress and found that the activity of PAL increased from 0-day of germination (0.155 µmol cinnamic acid/g) and reached a maximum on 5 day of germination (0.187 µmol cinnamic acid/g). The essential enzyme for the production of flavonoids and phenolics is PAL, which can catalyze the production of trans-cinnamic acid. The phenylpropanoid metabolic pathway can convert trans-cinnamic acid into an intermediary product such as coumaric acid and sinapic acid^50^.

The enzymatic activities of polyphenoloxidase (PPO), catalase (CAT) and peroxidase (POD) as antioxidant enzymes during the germination of wild mustard were screened in Fig. 1B. The PPO, CAT, and POD activities significantly increased (*P* < 0.01) and reached their highest values on 7-days sprouts (50 U/g), 3-days sprouts (450 U/g), and 5-days sprouts (500 U/g), respectively. The activities of three enzymes decreased gradually till 8-days of germination. The PPO, CAT and POD catalyzed the oxidation and generation of phenolic compounds and consumption of H_2_O_2_^51–54^.The highest activity of POD on 5-days sprouts indicated a high correlation between this enzyme and the highest phenolic and flavonoid levels on the same day. The POD is critical in the polymerization of mono- and di-phenols for the formation of polyphenols/phenolics and the activation of antioxidants during the germination process^40^.This may explain the association between the increase in POD enzyme activity and the increase in the concentration of polyphenols in 5-days mustard sprouts. Similarly, the phenolic content of green gram sprouts was associated with an increase in POD activity^55^. Several folds of increase in POD activity in 6-days sprouts of garden cress and 7-days sprouts of chia were demonstrated, which were strongly associated with high levels of polyphenols and antioxidant activity^18,19^. However, the findings indicated little association between the greatest phenolic and flavonoid levels on 5-days sprouts of mustard and the activities of PPO and CAT. In contrast, a strong correlation between the phenolic, flavonoid contents, the antioxidant activity, and the activities of PPO and CAT on 6-days sprouts of garden cress and 7-days sprouts of chia was detected^18,19^. In eight different barley cultivars after 24 h of germination, the PPO activity and total phenolic content increased together^56^. Collectively, there is a substantial correlation between the highest β-glucosidase and peroxidase activities, and highest phenolic and flavonoid levels, and maximum antioxidant activity on 5-days mustard sprouts.The reduction of PAL, PPO and CAT activities on 5-days sprouts may be attributed to the increase of the content of some phenolic compounds which act as enzymatic inhibitors^57,58^.

The antibacterial activity of wild mustard dry seeds and 5-days sprout phenolic extracts was investigated (Table 4).The inhibition zone diameters of dry seeds and 5-days sprouts against Gram-positive (*S. aureus*) strain were 5 and 17 mm, respectively, while the inhibition zones against Gram-negative (*E. coli*) were 4 and 16 mm, respectively. Gentamicin, as positive control, caused similar inhibition zones (14 and 16 mm) against the two tested pathogenic bacterial strains. Additionally, the MIC of dry seeds and 5-days sprouts against Gram-positive strain were 0.83 and 0.45 mg/ml, respectively, while the MIC against Gram-negative strain was 0.93 and 0.52 mg/ml, respectively. The MIC of Gentamicin against Gram-positive strain and Gram- negative strain were 0.64 and 0.92 mg/ml, respectively.These results indicated that the germination process of wild mustard seeds enhanced their antibacterial activity against the examined harmful bacterial strains. This may be attributed to enhancing all phenolic compounds such as *p*-hydroxybenzoic and sinapic acid and producing a new vanillic acid. These phenolic compounds may inhibit the growth of the bacteria through generating of H_2_O_2_ molecules, which caused changes in proteins of the bacteria and oxidative damage^59^. Similarly, the potent antibacterial activity of 7-days chia sprout phenolic extract was demonstrated toward the same bacteria^19^.

**Table 4:** Antibacterial activity and MIC of wild mustard seeds and 5-days sprouts.

Sample	*E. coli*		*S. A*	
	Inhibition zone (mm)	MIC (mg/mL)	Inhibition zone (mm)	MIC (mg/mL)
Mustard dry seed	4.0 ± 0.12^a^	0.93 ± 0.02^a^	5.0 ± 0.2^a^	0.83 ± 0.03^a^
Day 5-mustard sprouts	16.0 ± 0.8^b^	0.52 ± 0.01^b^	17.0 ± 0.7^b^	0.45 ± 0.03^b^
Gentamicin	14.0 ± 0.5^b^	0.92 ± 0.03^b^	16.0 ± 0.6^b^	0.64 ± 0.04^b^

Values in the same column with different superscripts (^a,b^) are significantly different at (*P* < 0.01).

In type 2 diabetes, the ability of insulin to stimulate cellular uptake of glucose from the blood is very low^60^. Thus, inhibitors of both α-amylase and α-glucosidase reduced the release of glucose from long-chain carbohydrates, followed by delaying glucose absorption and managing diabetes^61^. Acarbose is used as a positive control for inhibiting the activity of these enzymes^62^. The phenolic compounds of some plants showed antidiabetic activity^9^.The inhibitory effects of mustard seed and 5-days sprout phenolic extracts against α-amylase and α-glucosidase were evaluated (Table 5). IC_50_ values of mustard 5-days sprout phenolic extract for inhibition of α-amylase (82.6 µg GAE/ml) and α-glucosidase (39 µg GAE/ml) were less than those recorded for mustard seed extract (360 and 200 µg GAE/ml, respectively). The IC_50_ values of both mustard phenolic extracts for inhibition of α-glucosidase were lower than those for α-amylase. Acarabose had higher IC_50_ against both enzymes compared to both phenolic extracts. The results indicated that 5-days phenolic extracts showed strong inhibition for both enzymes compared to seed. This finding could be explained by the 5-days mustard sprout phenolic extract containing high levels of phenolic acids that possess potent antidiabetic properties, such as caffeic, *p*-hydroxybenzoic, and *p*-coumaric acids^63,64^.

**Table 5:** IC_50_ values of wild mustard seeds and 5-days sprouts phenolic extracts for inhibition of diabetic enzymatic activities.

Sample	IC_50_ (µg GAE/ml)	
	α-Amylase	α-Glucosidase
Mustard seeds	360 ± 12^a^	200 ± 8^a^
5-days sprouts	82.6 ± 3^b^	39 ± 1.2^b^
Acarabose	625 ± 22^a^	430 ± 15^a^

IC_50_ (µg GAE/ml) value is the phenolic concentration required to inhibit 50% of enzymatic activity. Values in the same column with different superscripts (^a,b^) are significantly different at (*P* < 0.01).

## Conclusions

The present study demonstrated that the phenolic and flavonoid contents increases several folds during the germination of wild mustard seeds. There is a substantial correlation between the highest β-glucosidase and peroxidase activities, and highest phenolic and flavonoid levels, and maximum antioxidant activity on 5-days mustard sprouts. The phenolic content of 5-days mustard sprouts showed potent antioxidant, antibacterial, and antidiabetic activities compared to dry seeds. Mustard sprouts are a rich source of antioxidant-phenolic compounds and could be used as functional food, antibacterial and antidiabetic agents.

## Data Availability

The datasets generated during and/or analyzed during the current study are available from the corresponding author upon reasonable request.
